# IMPACT OF DUAL SENSORY IMPAIRMENT ON HEALTH CARE INTERACTION

**DOI:** 10.1093/geroni/igz038.169

**Published:** 2019-11-08

**Authors:** Bonnielin Swenor, Bonnielin Swenor, Yasmeen Alshabasy, Emily Pedersen, Jennifer Deal, Amber Willink, Nicholas Reed

**Affiliations:** 1 The Wilmer Eye Institute, Johns Hopkins University School of Medicine, Baltimore, Maryland, United States; 2 The Wilmer Eye Institute, Johns Hopkins University School of Medicine, Department of Epidemiology; Johns Hopkins Bloomberg School of Public Health, Baltimore, Maryland, United States; 3 wesleyan University, Middletown, Connecticut, United States; 4 Cochlear Center for Hearing and Public Health, Johns Hopkins University Bloomberg School of Public Health, Baltimore, Maryland, United States; 5 Health Policy & Management and Cochlear Center for Hearing and Public Health, Johns Hopkins University Bloomberg School of Public Health, Baltimore, Maryland, United States

## Abstract

Using data from 46,029,364 Medicare beneficiaries included in the 2015 Current Beneficiary Survey (MCBS), we examined the relationship between dual sensory impairment (DSI) – concurrent vision impairment (VI) and hearing impairment (HI) – and accompaniment to physician visits. Analyses examined reasons for accompaniment and self-reported sensory impairment was categorized as: no sensory impairment (89%), hearing impairment (HI) only (5%), vision impairment only (4%), and DSI (1%). There was no difference in odds of accompaniment among HI compared to those without sensory impairment (odds ratio [OR] = 1.04; 95% Confidence Interval [CI]: 0.84,1.29); however, VI and DSI were associated with accompaniment: (OR=2.14; [CI]:1.6,2.8 and [OR]= 2.70; [CI]:1.55,4.72, respectively). Our study further demonstrates that older adults with sensory impairment are accompanied to physician visits more often than those without sensory impairment, and transportation is the most frequently reported reason for accompaniment among adults with VI and communication for those with HI.

